# How to Administer Chloroform

**Published:** 1872-12

**Authors:** 


*How to Administer Chloroform.*—Dr. G. Vivian Poore gives the following directions in the *London Lancet:*

"To administer chloroform, take a folded piece of lint of three or four thicknesses and of a size that can conveniently be held in the hollow of the fingers and palm, and on it pour about one drachm of chloroform. And here I may mention that, by holding the lint in the hand, evaporation from the upper surface of the lint is reduced to a minimum, and unnecessary waste of chloroform is prevented. This is another respect in which the piece of lint is superior to the folded towel. There is no use in accurately measuring the amount of chloroform poured on the lint, since the rate of evaporation is not materially different whether one drachm or more be used. Your patient being in a recumbent position, stand behind him, and before commencing the inhalation ascertain the condition of his pulse by placing the middle finger of the left hand on his left temporal artery, and while doing so endeavor to reassure him and allay any unnecessary fear on his part by a few cheerful words. See that all his clothes are loose. Then commence the inhalation, holding the piece of moistened lint three or four inches from the patient's face, and telling him to breathe quite naturally, always bearing in mind the danger of a strong atmosphere in the early stages, and carefully watching for coughing or acts of swallowing; when, if any such occur, the lint must be moved further away, and approached again more gradually. If the patient shows no signs of the chloroform being too strong, the lint may be gradually approached to within an inch and a half of the patient's mouth and nostrils; and then, to still more concentrate the atmosphere, the hand which holds the lint may be covered by one fold of an ordinary towel, which may hang loose over the mouth and chin, but should be so arranged above that the patient's eyes and forehead may remain visible. I believe the advice which has been given, 'Begin boldly; there is no risk with the first inhalations, and the patient may be instructed to draw full breaths,' to be as bad as advice can be. I would say, 'Begin cautiously, and tell the patient to breathe quite naturally.' Having got your patient fairly inhaling, continue the inhalation until there is no winking when the margins of the eyelids are touched, and the limbs are perfectly relaxed. You may then pronounce him ready for any operation. An inhalation generally takes about four minutes; often more, seldom less. You must bear in mind that, in the heat of summer, chloroform evaporates more rapidly than in the colder seasons of the year;

" While giving chloroform you must devote your undivided attention to the patient. Bear in mind that you are dealing with a potent poison, and that a state of complete anæsthesia may quickly become a state of death if the administrator is the least unmindful of the responsibility of his duties. Keep one finger on the pulse, and watch, as well as listen, very narrowly to the state of the respiration. The respiration is of more importance than the pulse undoubtedly, but the pulse is by no means to be neglected. It often gives most important information as to the state of the patient, and it is so easily felt at the temporal artery by the left hand, which if not engaged in this way would be doing nothing, that it seems to me not wise to advise that the state of the pulse should be entirely neglected; besides the anæsthetist should always be ready with his answer when interrogated by the surgeon, as he often will be, as to the condition of the patient. When the patient becomes excited he must be watched very narrowly, and if the respirations become proportionately quickened so must the chloroform atmosphere be proportionately weakened by withdrawing the hand slightly from the face. When the muscles become rigid and the patient holds his breath and becomes livid, the chloroform should be given very weak indeed, because the patient is becoming semi-asphyxiated by the suspension of respiration, and this condition is often followed by extreme rapidity and depth of respiration, and a dangerous condition is apt to supervene since anæsthesia is added to asphyxia. Here I may, I think, fittingly mention one very important fact about chloroform-vapor—namely, its cumulative action. This should always be borne in mind, for the state of narcosis frequently intensifies for half a minute or more after the suspension of the administration. This is of course due to the entry into the blood of the chloroform-vapor which was in the lungs when the administration cease

